# A statistical atlas of cerebral arteries generated using multi-center MRA datasets from healthy subjects

**DOI:** 10.1038/s41597-019-0034-5

**Published:** 2019-04-11

**Authors:** Pauline Mouches, Nils D. Forkert

**Affiliations:** 1 Department of Radiology, University of Calgary, Calgary, Canada; 2 Hotchkiss Brain Institute, University of Calgary, Calgary, Canada

**Keywords:** Magnetic resonance imaging, Anatomy, Diagnostic markers, Cardiovascular diseases, Brain imaging

## Abstract

Magnetic resonance angiography (MRA) can capture the variation of cerebral arteries with high spatial resolution. These measurements include valuable information about the morphology, geometry, and density of brain arteries, which may be useful to identify risk factors for cerebrovascular and neurological diseases at an early time point. However, this requires knowledge about the distribution and morphology of vessels in healthy subjects. The statistical arterial brain atlas described in this work is a free and public neuroimaging resource that can be used to identify vascular morphological changes. The atlas was generated based on 544 freely available multi-center MRA and T1-weighted MRI datasets. The arteries were automatically segmented in each MRA dataset and used for vessel radius quantification. The binary segmentation and vessel size information were non-linearly registered to the MNI brain atlas using the T1-weighted MRI datasets to construct atlases of artery occurrence probability, mean artery radius, and artery radius standard deviation. This public neuroimaging resource improves the understanding of the distribution and size of arteries in the healthy human brain.

## Abstract (summary)

**Table:** 

Design Type(s)	feature extraction objective • anatomical image analysis objective • data integration objective
Measurement Type(s)	brain blood vessel
Technology Type(s)	MRI-Based Angiogram
Factor Type(s)	age • ethnic group • sex
Sample Characteristic(s)	Homo sapiens • brain

Machine-accessible metadata file describing the reported data (ISA-Tab format)

## Background & Summary

The human cerebroarterial system is composed of a complex system of arteries that supply the brain cells with oxygen and nutrition. Cerebroarterial networks are difficult to analyze due to their composition of small and large arteries, which have a complex organization of branches and cyclic connections that varies among individuals.

Analyzing cerebroarterial structures could aid diagnosis and research of multiple cerebrovascular diseases, such as stenoses, aneurysms, arteriovenous malformations, or ischemic strokes, all of which are related to alterations of the arterial system. Some changes in the cerebrovascular system could also prove valuable as early biomarkers for neurological diseases, such as Alzheimer’s disease^1^. However, detecting structural arterial abnormalities requires detailed knowledge of the normal morphology and distribution of the cerebral arteries in the brain.

Most brain atlas studies have focused on representing and describing the location of tissue types, and anatomical and functional brain regions, while artery morphology and distribution within the brain are investigated rather seldomly for this purpose. A potential reason for this lack is that a large dataset is required to fully capture the variability of these small structures in a healthy population. Cerebroarterial atlases would help to describe the normative data of healthy patients and enable the detection of abnormalities in patients with diseases, potentially even at an early stage.

Magnetic resonance angiography (MRA) is an imaging technique, which enables the visualization of the cerebral arteries and has been used in a limited number of previous studies that focused on generating statistical arterial atlases using datasets from healthy subjects. However, these studies have only used a very limited number of datasets (n = 9^2^, and n = 54^3^), which might not capture the full range of the inter-individual variability given the small structure of interest. Another previously described study in this context used 700 datasets but employed only linear registration for spatial normalization to the atlas space^4^. Arteries are often embedded within the sulci of the brain. Although the general topology of the major sulci is similar between subjects, a linear registration is not able to account for the fine inter-subject differences regarding the exact location and size of the sulci. For this reason, a linear registration falsely misaligns and therefore blurs the arteries around the sulci in the atlas space. Finally, another recently published probabilistic vessel atlas used a total of 167 MRA datasets from healthy subjects for atlas generation using non-linear registration^5^. However, all subjects are within a rather limited age range of 64–68 years, which might limit its potential to identify vessel changes in younger subjects. Finally, all of the aforementioned studies only used single center, single scanner datasets, which ignores potential variations caused by the imaging setup, e.g. the scanner, field strength, coil, and sequence parameters.

The aim of this work was to generate a free and publicly available statistical cerebroarterial atlas using a dataset large enough to cover the inter-individual variability, also across age. More precisely, this atlas was generated using 3D time-of-flight (TOF) MRA images of healthy subjects, acquired at different centers with a variety of scanners. The TOF MRA is a particularly useful imaging modality for this purpose as it typically does not require any contrast agent and offers a good blood-to-background contrast of arteries. The TOF MRA sequence provides a high level of anatomical information, which is needed to evaluate the complex vascular network and enables the detection of cerebrovascular diseases^6^. The use of multi-center data acquired from healthy adults across the whole age spectrum allows a better representation of the true variability of the arteries in the brain and makes it more compatible with any other new dataset.

**Fig. 1:**
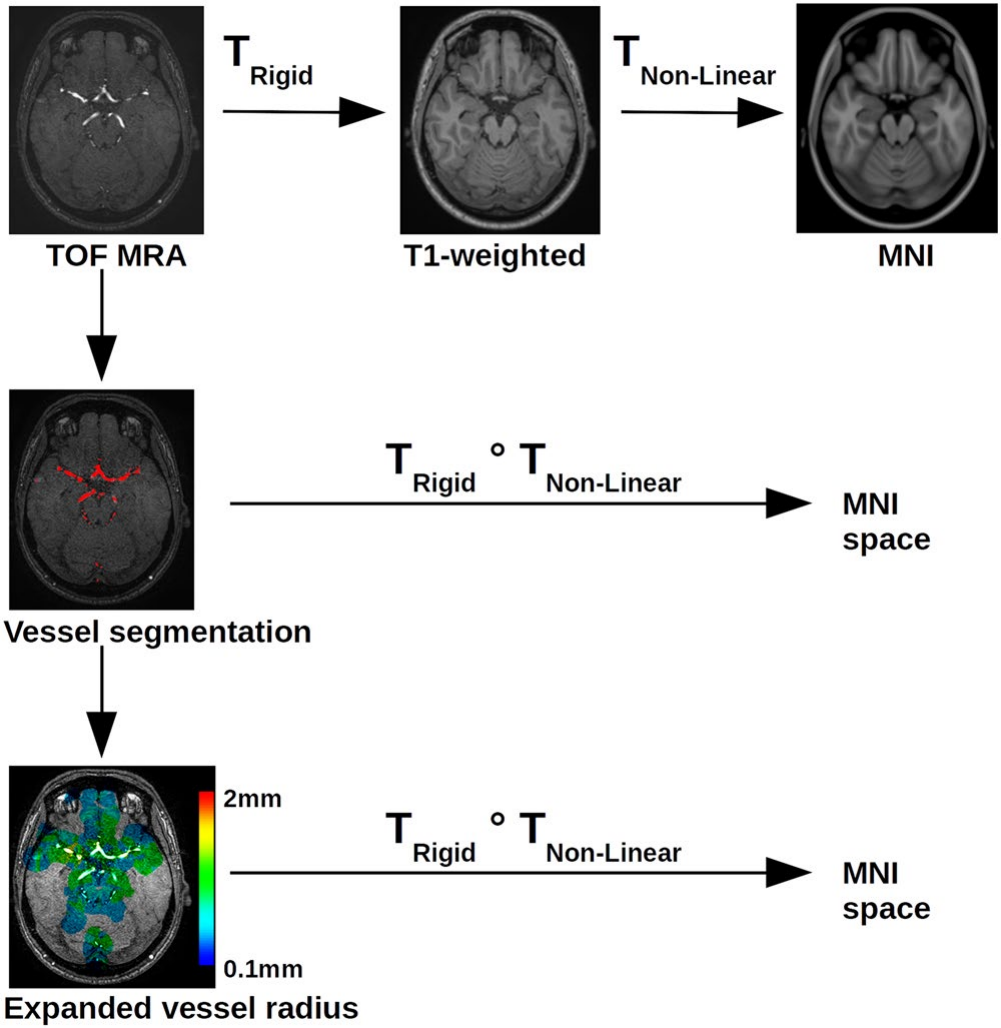
Image processing pipeline of the statistical atlas generation.

Figure 1 shows a diagram of the image processing pipeline used for this research, described in more detail in the following.

## Methods

### Datasets

A total of 603 datasets, including high-resolution T1-weighted MRI and TOF MRA datasets, from normal, healthy subjects were available for the generation of the statistical cerebroarterial atlas described in this work.

494 of these datasets from healthy subjects were downloaded from the IXI database (http://brain-development.org), which combines MRI acquisitions from three different centers in London, UK. For this work, only the datasets from the Hammersmith Hospital (IXI-HH) and the Guy’s Hospital (IXI-Guys) were used. The magnetic resonance angiographies from the third center, the Institute of Psychiatry, did not appear to be conventional TOF MRA datasets and the scan parameters were not available for this center for further investigations. The IXI database reports that all subjects are normal healthy. Unfortunately, the exact definition of normal healthy is not described. However, as the goal of this database was specifically to collect only data from healthy subjects, it should be reasonable to assume that all subjects were properly screened and diagnosed healthy given common criteria.

In addition to this, the remaining 109 MR brain images from healthy volunteers used for the artery atlas generation were collected and made available by the CASILab at The University of North Carolina at Chapel Hill (UNC) and distributed by the MIDAS Data Server at Kitware, Inc. (http://insight-journal.org/midas/community/view/21). The UNC database specifies that only subjects with a normal appearing brain MRI and MRA were included. Patients with a history of diabetes, hypertension, head trauma, psychiatric disease, or other symptoms affecting the brain were excluded.

After further analysis of the associated clinical data, ten subjects from the IXI database and ten subjects from the UNC database were excluded because of missing clinical data while seven additional UNC subjects were excluded because of other incidental findings (e.g. ethmoid polyp, tiny intracanalicular acoustic tumor, epidermoid cyst, and a tiny stroke) even though reported not to affect the brain and vascular anatomy.

After pre-processing (see below), 32 additional datasets were removed because of insufficient data quality or image processing results. The demographic information of the remaining 544 datasets, used for the atlas generation, are described in Table 1. It can be seen that the age and sex distributions were similar for all three centers.

**Table 1:** Demographic information of the finally included subjects.

Center	n	Sex		Mean Age (range)	Ethnicity				
		M	F		White	Asian	Black	Hispanic	Other
IXI-HH	175	86	89	47 (20–82)	129	25	8	8	5
IXI-Guys	282	122	160	50 (20–86)	239	24	3	1	15
UNC	87	40	47	42 (19–68)	75	8	4	0	0
Total	544	248	296	47 (19–86)	443	57	15	9	20

Multiple MR scanners and imaging parameters were used for the acquisition of these datasets in the three different centers. The scanners included an Intera 3T MRI scanner (Philips Medical Systems, Best, The Netherlands) used by the Hammersmith Hospital (IXI-HH), London, UK, a Gyroscan Intera 1.5T (Philips Medical Systems, Best, The Netherlands) used by Guy’s Hospital (IXI-Guys), London, UK, and an Allegra 3T MR scanner (Siemens Medical Systems Inc., Germany) used for data acquisition at The University of North Carolina at Chapel Hill (UNC).

The detailed scanning parameters can be found in the respective online sources where the data was downloaded while the most important TOF MRA scanning parameters are given in Table 2. The spatial resolution of the MRA and T1-weighted MRI datasets was mostly comparable for the different centers. More precisely, all T1-weighted MRI datasets exhibited an in-slice resolution of ~1.0 mm in both directions and slice thickness of ~1.2 mm except for the UNC datasets, which were acquired with a slice thickness of 1.0 mm. The spatial resolution of all TOF MRA datasets was comparable between the centers while the other main TOF MRA acquisition parameters differed considerably.

**Table 2:** TOF MRA acquisition parameters.

	Resolution (mm^3^)	Echo time (ms)	Repetition time (ms)	Flip angle (°)
IXI-HH	0.47 × 0.47 × 0.8	5.75	16.7	16
IXI-Guys	0.47 × 0.47 × 0.8	6.9	20	25
UNC	0.51 × 0.51 × 0.8	3.56	35	22

### Pre-processing

The pre-processing of the data consisted of four steps. In the first step, one time-of-flight MRA dataset with good blood-to-background contrast and without any obvious imaging artefacts was manually selected from the UNC database. This dataset was then used as the reference for intensity normalization of all other datasets using the linear histogram matching method described by Nyul *et al*.^7^, with 100 histogram bins and 25 matched quantile values, implemented in the Insight Segmentation and Registration Toolkit (ITK)^8^. This was necessary to correct for the considerable intensity differences between the TOF MRA datasets acquired in the different centers resulting from the different image acquisition parameters.

In the second step, a vesselness filter was used for vessel enhancement^9^. After combination of the resulting vesselness parameter image with the TOF MRA intensity image using a fuzzy logic approach^10^, a level-set segmentation approach with anisotropic energy weights^11^ was used for extraction of the individual cerebroarterial vessel system using the described parameter values.

The extracted artery segmentation was then used for estimation of the artery radius at each voxel in the third step. Briefly described, the 3D centerline representation was calculated for each segmentation using the method described by Lee *et al*.^12^. After this, the distance of each centerline voxel to the closest non-vessel voxel was computed using the distance transform described by Danielsson^13^, which was used to define the radius for each centerline voxel. A second distance transform was then used to identify the closest centerline voxel for every voxel within 5 mm distance to a centerline voxel. By doing so, the radius value from the centerline voxel as determined by the first distance transform was assigned to all non-centerline voxels closest to this centerline voxel and within 5 mm distance. Practically, this operation expands each artery to a radius of 5 mm. However, the artery radius information associated with each voxel as extracted from the original artery segmentation is not altered by this. In other words, this operation only dilates the artery boundaries while keeping the original radius information unchanged. Using this advanced distance transform approach described above in contrast to a simple dilation, ensures that the vessel radius is not smoothed across arteries in close proximity and also not along an artery so that tampering of an artery is not affected. The primary motivation of this step was to increase the statistical power calculating the average artery radius for very small artery segments that can be associated with vessel probabilities far below 1%. Thus, by expanding the individual artery calibre maps, more artery calibre information will be available at each voxel in the atlas space for calculating the artery atlas described below.

Finally, each individual TOF MRA sequence as well as the corresponding artery segmentation was spatially normalized to the 0.5 mm^3^ isotropic Montreal Neurological Institute (MNI) brain atlas^14^ reference space. The 0.5 mm^3^ isotropic atlas was used since the TOF MRA datasets were acquired with 0.5 mm^2^ in-slice resolution. The different reference coordinates between the scanners used to acquire the TOF MRA datasets and varying patient positions inside the scanners result in non-aligned TOF MRA datasets. In order to generate the statistical atlas, the intensity-normalized TOF MRA datasets, the corresponding artery segmentations, and thickness maps (average and standard deviation) must be represented in a common reference space. The registration required for this was performed in two steps. The first step consisted of registering each TOF MRA dataset to the corresponding T1-weighted MRI dataset. As both images are from the same patient, a rigid transformation was used for this purpose. This rigid registration was performed using the block-matching approach described by Ourselin *et al*.^15^ implemented in the NiftyReg software package^16^ using default parameters. After this, the T1-weighted MRI datasets were registered to the MNI brain atlas. This registration was performed using a free-form deformation as implemented in the NiftyReg software package^16^ using the default parameters, after initialization with an affine registration using the same approach as used for the rigid registration described above. Finally, the two transformations, rigid and non-linear, were concatenated to transform the TOF MRA dataset and the corresponding artery information directly into the reference space of the MNI brain atlas without double interpolation errors. A nearest-neighbour interpolation was used in all cases to prevent false smoothing of the vessel information at the artery edges in the transformed segmentation and artery radius datasets while a linear interpolation was used for transformation of the intensity-normalized TOF MRA datasets into atlas space.

### Atlas generation

**Fig. 2:**
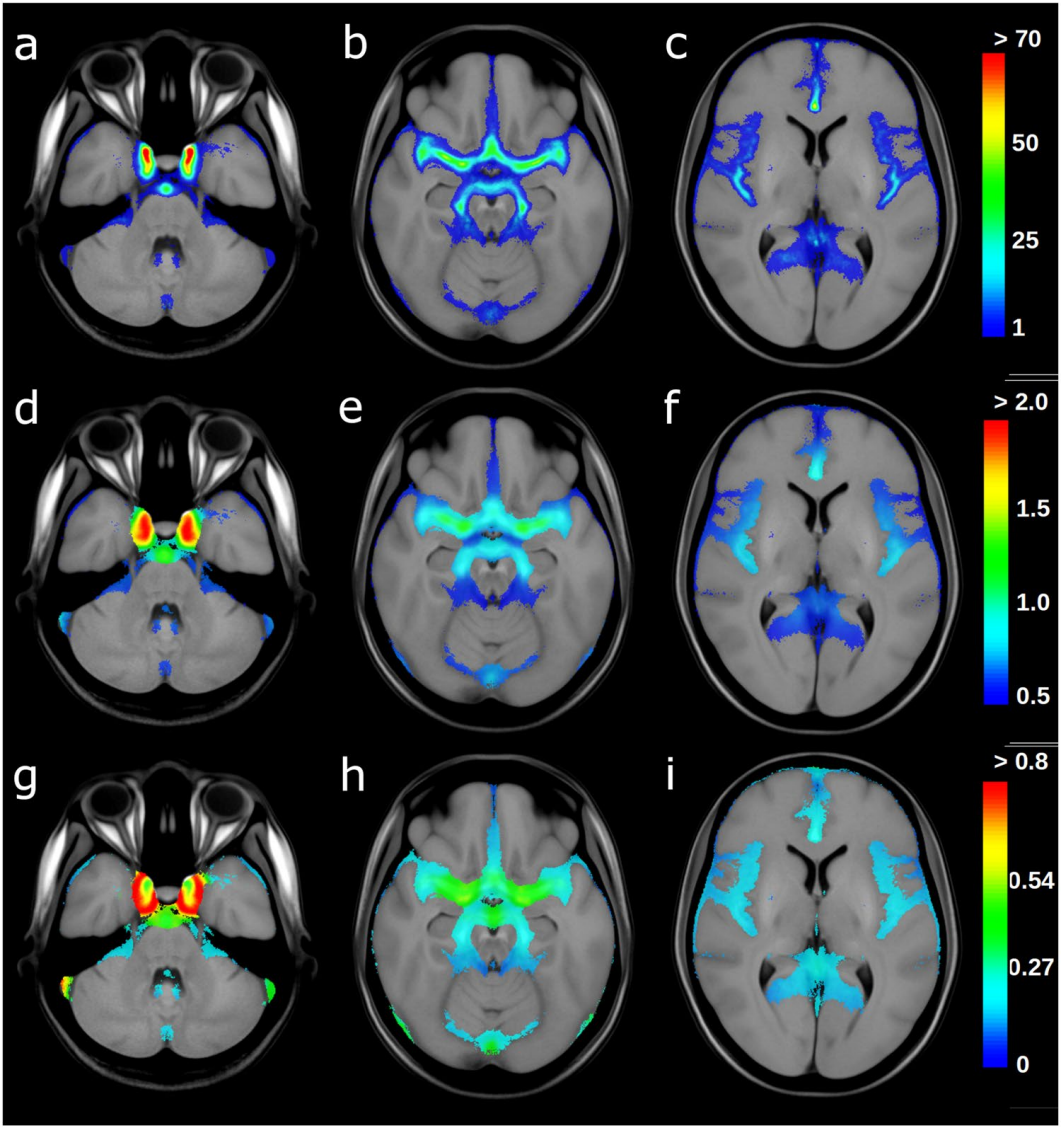
Cerebroarterial statistical atlas. Selected slices from the artery probability atlas (**a**–**c**) the mean artery radius atlas (**d**–**f**) and the standard deviation atlas (**g**–**i**) overlaid on the TOF MRA average atlas. The artery probabilities are shown in percent while the mean radius and its standard deviation are showed in mm.

A total of four atlases datasets were generated using the available registered data. This includes a TOF MRA average atlas as well as corresponding statistical maps representing the regional artery probability, mean artery radius, and standard deviation of the artery radius (Fig. 2).

The registered TOF MRA datasets were first used to create an average TOF MRA intensity atlas. For each voxel, the intensity of the voxels in the atlas represents the mean intensity of the corresponding voxels in all registered TOF MRA datasets accounting for different anatomical brain coverages in the individual datasets. This means that voxels not covered by an individual TOF MRA datasets did not influence the average calculation.

An artery probability atlas was generated using the registered artery segmentations (not dilated), by averaging the artery segmentations of all patients, again accounting for different volumes covered by the individual TOF MRA datasets. A manually defined brain mask, which also includes arteries at the border of the brain as well as the internal carotid artery and basilar artery segments outside the brain was finally used to remove the border artefacts originating from inaccurate registrations or vessel segmentations, e.g. within the bone marrow or optic nerve. This atlas represents the probability of an artery occurrence at each voxel location in the atlas. Since the UNC datasets display parts of the venous sinus, which are not visible in the other datasets, these areas were manually removed from the generated probability atlas to prevent false artery probability metrics in these areas.

After this, the registered artery radius datasets were used to compute the mean artery radius and corresponding standard deviation for each voxel of the atlas. The mean artery radius atlas was computed for each voxel by adding all non-zero values in the registered expanded artery radius datasets and dividing the result by the number of non-zero voxels. Finally, the atlas was masked by the brain mask described above.

In addition to the simple averages, the standard deviation of the artery radius was also computed for each voxel by adding the squared differences of the artery radius in the registered expanded vessel radius map and the corresponding value in the average artery radius atlas map and subsequent division by the number of non-zero voxels.

### Z-scores

The statistical atlas allows calculating a z-score for each artery voxel of a new datasets. The z-score value quantifies the distance of a new observation from the normative average. This allows analyzing TOF MRA datasets of new patients to identify variations from the population average regarding the radius for each artery voxel. Therefore, the arteries of a new TOF MRA dataset must be segmented and the corresponding artery radius has to be calculated. After non-linear registration of the TOF MRA average intensity atlas to the patient-specific TOF MRA dataset and transformation of the corresponding average artery radius and standard deviation map, the z-score can be calculated for each segmented voxel by:

1 $${Z}_{i}=\frac{{x}_{i}-{\mu }_{i}}{{\sigma }_{i}}.$$

Here, *Z*_*i*_ denotes the z-score, whereas *x*_*i*_ denotes the radius of the segmented artery in the patient-specific TOF MRA dataset, and *μ*_*i*_ and *σ*_*i*_ the corresponding mean and standard deviation of the artery radius in the probabilistic vessel atlas for each voxel i.

## Data Records

The statistical atlas consists of four image files in the nifti format and in MNI reference space (0.5 mm^3^)^17^. These files include the TOF MRA average atlas (tofAverage.nii.gz), the vessel probability atlas (vesselProbabilities.nii.gz [in %]), the mean artery radius atlas (vesselRadius.nii.gz [in mm]), and the standard deviation of the artery radius atlas (vesselRadiusStd.nii.gz [in mm]). All images are saved using float values. The TOF MRA and T1-weighted datasets used for atlas generation can be downloaded from the original sources.

## Technical Validation

### Datasets

Every dataset and the corresponding results of the processing steps, i.e. the artery segmentation and non-linear registration approach were visual inspected to ensure proper quality for the atlas generation. Therefore, the artery segmentations were visualized in the orthogonal slices as well as using 3D volume rendering of the whole extracted vessel network. Datasets were excluded in case of motion artefacts, other imaging artefacts preventing a proper vessel segmentation such as severe ringing artefacts, pulsation artefacts, flow-dependent signal cancellations, as well as noise artefacts resulting in a considerable over or under-segmentation of the arterial system leading to artery segmentations differing from the expected normal variation of the vascular network. However, some of the TOF MRA datasets used for atlas generation still showed minor artefacts not considerably impacting the artery segmentation.

Likewise, the quality of the registration was visually checked for every dataset using synchronized viewing of the two datasets (MNI atlas and registered TOF MRA dataset) in the orthogonal views as well as checkerboard display of the two datasets. Datasets were excluded due to considerable mis-registrations.

A total of two datasets were excluded due to severe imaging artefacts, and 30 datasets due to poor image quality leading to bad segmentation results. None of the datasets had to be removed because of unsatisfying registration results. Thus, a total of 544 datasets of the 576 available datasets were finally used for atlas generation.

### Statistical atlas

The generated statistical cerebroarterial atlas was visually and quantitatively inspected to evaluate its consistency with previous findings in literature, mostly based on cadaver studies.

The atlas clearly displays the main arteries, represented by high occurrence probabilities (Fig. 2). Generally, the probabilistic artery atlas shows high artery occurrence probabilities for large arteries, which are decreasing as the vessel radius decreases, which is in line with previous findings^4^.

**Fig. 3:**
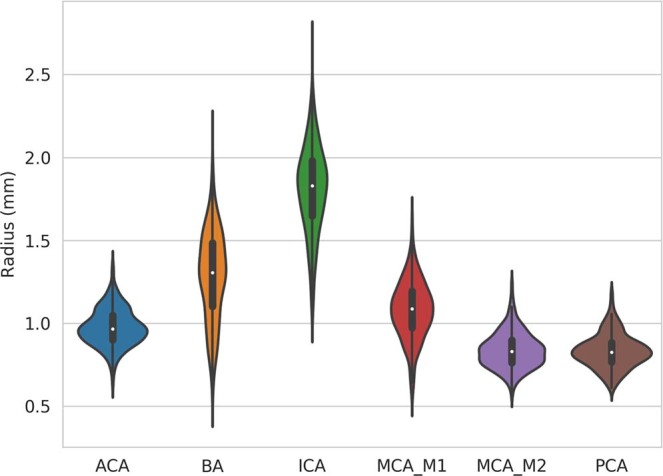
Violin plots of the mean vessels’ radius of the major brain arteries across patients. ACA: anterior cerebral artery; BA: basilar artery; ICA: internal carotid artery; MCA_M1: middle cerebral artery, M1 segment; MCA_M2: middle cerebral artery, M2 segment; PCA: posterior cerebral artery.

For quantitative analysis, small regions-of-interest were manually defined within the major arteries in the generated average TOF MRA atlas and used to determine the average radius values of these arteries in each individual subject. Figure 3 shows the corresponding violin plots for the major arteries in the brain across the subjects used for atlas generation. Overall, it can be seen that the average radius estimates are normally distributed for all arteries analyzed while the plots also suggest that there are no severe outliers.

**Fig. 4:**
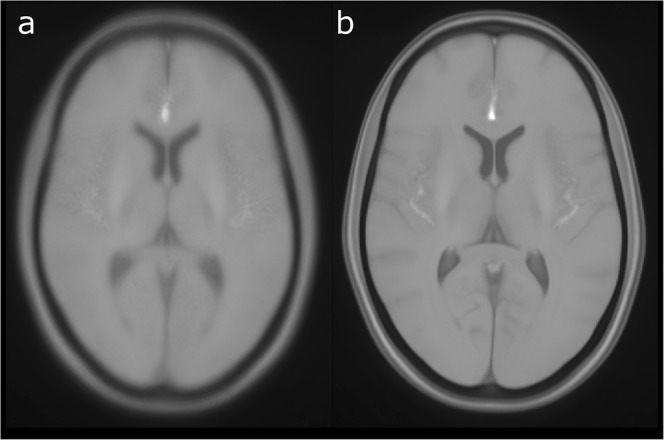
Average TOF MRA intensity atlas. (**a**) Generated using linear registration, (**b**) generated using non-linear registration.

The highest artery occurrence probability is located at the base of the internal carotid artery (ICA) with an occurrence probability of up to 90% and a mean vessel radius of 1.8 mm, which is within the range of 1.25 to 3.5 mm previously reported by Saeki and Rhoton^18^. The internal carotid artery bifurcates into the middle cerebral artery (MCA) and anterior cerebral artery (ACA). The anterior cerebral artery displays a mean radius of around 1 mm and an occurrence probability up to 55% in the artery atlas. Similarly, previous research suggest that the radius of the anterior cerebral artery is ranging from 0.45 to 2 mm^19^. The M1 and M2 (insular arteries) segments of the middle cerebral artery display a mean radius in the generated artery atlas of up to 1.2 mm and 0.8 mm, respectively, and a probability occurrence up to 45% and 30%, respectively. The average radius values for these arteries as determined by the probabilistic artery atlas are at the lower end of the range reported in literature based on human cadaver brain studies (M1 segment radius in the range of 1.3 to 2 mm^20^, and 1.2 to 2.2 mm^21^; M2 segment diameter ranging from 0.8 to 1.5 mm^20^).

The basilar artery (BA) is mainly responsible for the posterior cerebral circulation and bifurcates into the posterior cerebral arteries (PCA). The basilar artery displays an occurrence probability of up to 45% and an average radius of up to 1.35 mm in the generated artery atlas, which is slightly smaller than the average of 1.6 mm reported by Smoker *et al*.^22^. However, Smoker *et al*. also reported that the thickness of the basilar artery is highly variable with the radius ranging from 0.8 mm to 3 mm in healthy subjects. This considerable radius variation is also evident in the corresponding violin plot shown in Fig. 3. The posterior cerebral arteries display an occurrence probability of up to 30% and a mean vessel radius of up to 0.80 mm, which is coherent with the previously described values of cadaver studies which reported a radius between 0.4 and 1.9 mm^23^ in the P1 segment.

Overall, the radius measurements of the arteries are rather at the lower end of the corresponding ranges described in literature based on cadaver studies. This underestimation is most likely caused by the fact that cadaver studies typically measure the arteries from the outside, which includes the vessel wall, while magnetic resonance angiography only measures the inner lumen.

Finally, comparing the average TOF MRA intensity atlas generated using non-linear registration to the corresponding atlas generated using linear registration only, clearly displays the benefit of using non-linear registration for this purpose as the resulting atlas displays sharper edges (Fig. 4). The non-linear registration permits elastic deformations, which are necessary to correct for morphological differences between subjects and allows generating a crisper average atlas. Thus, the average TOF MRA intensity atlas generated using non-linear registration could be used for direct registration of individual TOF MRA datasets without using T1-weighted MRI datasets in an intermediate registration step.

### Z-scores evaluation

**Fig. 5:**
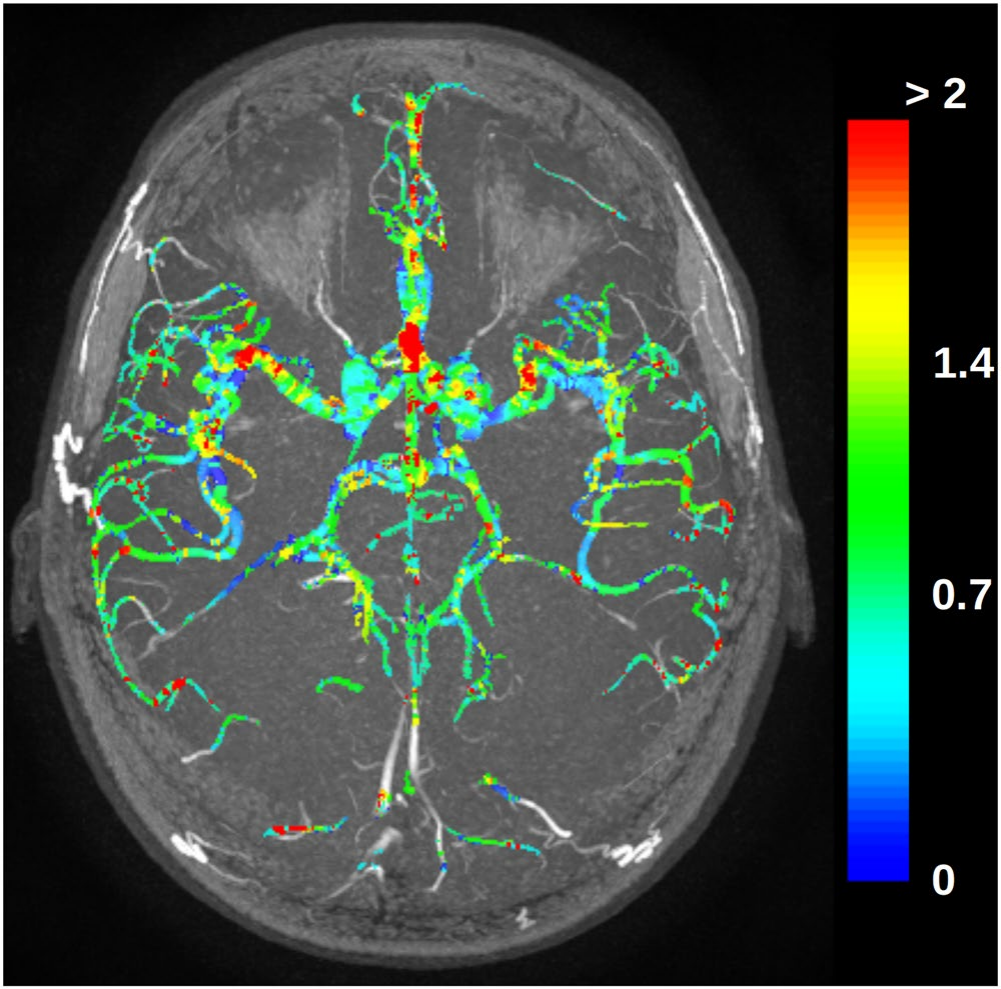
Maximum intensity projection of the absolute values of the voxel by voxel z-scores of a dataset overlaid on the maximum intensity projection of the TOF MRA.

Figure 5 shows a maximum intensity projection visualization of an individual artery segmentation of a dataset not used for the atlas generation with the color-coded absolute z-scores. The high z-scores, in red, indicate regions where the individual cerebroarterial system varies the most from the population average. As a healthy subject was used for this experiment, it is expected that there are only a few, potentially noise-related, high z-scores. This experiment shows that z-scores values might be valuable to detect vessel abnormalities.

## Usage Notes

The NIfTI data format allows using the atlas data in tools used for neuroimage analysis such as Slicer, FreeSurfer, or FSL. Multiple freely available tools can be used for registration such as NiftyReg used in this work or Advanced Normalization Tools (ANTs). For artery segmentation and analysis in individual TOF MRA datasets, freely available software tools such as the Vascular Modeling Toolkit could be used.

## ISA-Tab metadata file

Download metadata file

## Data Availability

The image registration tools used in this work are freely available in the NiftyReg toolkit. The vessel segmentation method is not freely available but can be replaced by any freely available software tools such as the Vascular Modeling Toolkit.
